# Supplementary material for: CACE closed: A multiverse examination of the influence of implementation variability on student outcomes in a randomised controlled trial of a universal, school-based social-emotional learning intervention
Source: PLoS One. 2026 Jun 2;21(6):e0349949. doi: 10.1371/journal.pone.0349949 (PMC13229310; doi:10.1371/journal.pone.0349949)
Supplement: S1 File — (DOCX) [file pone.0349949.s001.docx]

**CACE closed: A multiverse examination of the influence of implementation variability on student outcomes in a randomised controlled trial of a universal, school-based social-emotional learning intervention**

Dosage

| **Item** | **Response format** | |
| --- | --- | --- |
| Did you teach Session 0: Beginning the adventure? | No | Yes |
| Did you teach Session 1: Valuing our differences and similarities | No | Yes |
| Did you teach Session 2: Understanding and expressing our emotions | No | Yes |
| Did you teach Session 3: Recognising other people’s feelings | No | Yes |
| Did you teach Session 4: Helping each other and coping skills | No | Yes |
| Did you teach Session 5: Friendship | No | Yes |
| Did you teach Session 6: Challenges in friendship | No | Yes |
| Did you teach Session 7: Dealing with frustration | No | Yes |
| Did you teach Session 8: Dealing with stress | No | Yes |
| Did you teach Session 9: Dealing with conflict | No | Yes |
| Did you teach Session 10: Unfairness in daily life | No | Yes |
| Did you teach Session 11: Dealing with bullying | No | Yes |
| Did you teach Session 12: Unfairness and injustice in the world | No | Yes |
| Did you teach Session 13: Coping with change | No | Yes |
| Did you teach Session 14: Coping with loss | No | Yes |
| Did you teach Session 15: Helping others in difficult situations | No | Yes |
| Did you teach Session 16: Let’s review | No | Yes |
| Did you teach Session 17: Celebration | No | Yes |

Adherence

For the following items, we want you to think about your use of the Passport guidance materials when teaching the above lessons:

| **Item** | **Response format** |
| --- | --- |
| I covered the key goals/objectives of Passport lessons that I taught | Slider, 0-100% |
| I followed the structure and sequence of activities outlined in the guidance materials for Passport lessons that I taught | Slider, 0-100% |
| I adhered to the guidance materials when teaching the core activities of Passport lessons | Slider, 0-100% |

Quality

For the following items, we want you to think about how you felt about teaching the above lessons:

| **Item** | **Response format** |
| --- | --- |
| I felt able to clearly explain key activities to children when teaching Passport lessons | Slider, 0-100% |
| I felt able to respond to the needs of children during Passport activities | Slider, 0-100% |
| I felt able to engage the pupils in my class during Passport lessons | Slider, 0-100% |
| I felt well prepared to teach Passport lessons | Slider, 0-100% |

Responsiveness

For the following items, we want you to think about how children in your class responded when you were teaching the above lessons:

| **Item** | **Response format** |
| --- | --- |
| Children in my class actively participated (e.g., initiated and elaborated on discussion topics) in Passport | Slider, 0-100% |
| Children in my class engaged (e.g., completed activities with interest) with Passport | Slider, 0-100% |
| Children in my class showed interest and enthusiasm (e.g., looked forward to lessons, asked/reminded you about them) for Passport | Slider, 0-100% |
| Children in my class enjoyed (e.g., discussed lessons positively) taking part in Passport | Slider, 0-100% |

Reach

For the following item, we want you to think about who was present when you were teaching the above lessons:

| **Item** | **Response format** |
| --- | --- |
| What proportion of your class that were present, on average, during Passport lessons? *Consider withdrawal for additional support etc.* | Slider, 0-100% |
