# Supplementary material for: CACE closed: A multiverse examination of the influence of implementation variability on student outcomes in a randomised controlled trial of a universal, school-based social-emotional learning intervention
Source: PLoS One. 2026 Jun 2;21(6):e0349949. doi: 10.1371/journal.pone.0349949 (PMC13229310; doi:10.1371/journal.pone.0349949)
Supplement: S2 File — (DOCX) [file pone.0349949.s002.docx]

**CACE closed: A multiverse examination of the influence of implementation variability on student outcomes in a randomised controlled trial of a universal, school-based social-emotional learning intervention**

There is no explicit guidance in Steegen and colleagues’ [1] paper on how to identify arbitrary decisions, except that decisions should be “sound and justifiable” (p.703). Simonsohn et al. [2, 3] further specify that arbitrary decisions are those that lack theoretical justification, such that choices with theoretical rationale are not classified as arbitrary [4]. The multiverse should be compromised of truly arbitrary decisions and not methodologically inferior specifications [3] that would “quickly explode the size of the multiverse” [4] (p.4). Del Giudice and Gangestad [4] call for researchers to critically consider why and how decisions were classified as arbitrary to avoid nonsensically inflating the multiverse space and taking attention away from potentially important findings.

In reporting our construction of the multiverse of data specifications, we follow systematic procedures and evaluation tools developed by Short et al. [5] and Del Giudice and Gangestad [4]. Following identification of these decision pathways in our CACE model, we assessed decision equivalency. We were guided by Del Giudice and Gangestad [4] who defined three outcomes when assessing the equivalency:

1. Type N decision: when one decision is considered theoretically superior to another, the inferior decision is removed from the multiverse dataset.
2. Type E decision: when alternative defensible options are deemed truly equivalent, then all options are included in the multiverse dataset.
3. Type U decision: when the equivalence of alternative options is ambiguous, then the exploratory nature of their inclusion in the multiverse should be transparently reported.

We identified four defensible decision pathways in our CACE model:

**Dependent variables.** This study uses secondary data from a large randomised trial of the Passport programme [6]. The pool of available dependent variables in our secondary dataset included: internalising symptoms, well-being, emotional regulation, peer social support, loneliness, peer victimisation, health-related quality of life and academic attainment. We chose peer social support, loneliness and peer victimisation as we were interested in examining the impact of Passport on students’ social relationships. The other available variables were therefore not theoretically relevant. This was therefore classified as a Type N decision.

**Compliance variable.** A teacher self-report implementation survey captured data on dosage, fidelity, reach, quality and responsiveness. The data captured from this measure provided five options for a compliance variable. Recent systematic reviews reveal how each of these implementation dimensions are associated with student outcomes [7, 8]. These findings informed the decision to consider each implementation dimensions as a potential marker of intervention compliance in our CACE models. This was therefore classified as a type U decision.

**Compliance threshold.** CACE is limited by its reliance on a binary marker of compliance because defining 'full' compliance of a prevention intervention is significantly challenging. CACE is dependent on the exclusion restriction assumption (non-compliers derive no benefit from the intervention) which is difficult to validate in the absence of theory-informed compliance thresholds (Education Endowment Foundation, 2018) which are largely untested and unknown in psychological and educational interventions (Durlak, 2010). Accordingly, Peugh and colleagues [9] recommend: "*a series of additional sensitivity analyses could be conducted using several binary compliance indicator (“U”) variables defined at multiple cut-points (e.g., 50%, 70%, 90%, 95%) and observing the change in the CACE treatment effect estimate”* (p.23).

It is possible to conduct multiple sensitivity analyses to explore a broader range of compliance thresholds. We made the decision to pre-registered a sensitivity analysis examining two compliance thresholds (50^th^ and 75^th^ percentile). This choice was based on established practice in educational research, as many CACE studies in education utilise these two thresholds [10, 11, 12, 13, 14, 15], therefore allowing comparison with other intervention studies using identical thresholds. This decision was also pragmatic as each CACE model requires substantial computational time (>24 hours). This was therefore classified as a type U decision.

**Compliance predictors.** Available compliance predictors were identified from the secondary dataset. These included school-level variables (e.g., school size, % free school meal eligibility), teacher-level variables (e.g., perceived stress, attitudes towards SEL) and student-level variables (e.g., ethnicity, baseline scores). A collaborative ‘crowdsourcing’ approach across the research team informed selection. Student-level covariates were selected based on theory and evidence that contextual factors (e.g., student characteristics and perceived need) can influence class climate and teacher compliance behaviours [16], such that children with greater perceived needs can increase [e.g., 17] or impede [18] programme delivery. Selection of teacher-level covariates was informed by evidence of a relationship between perception of the school’s SEL culture and use of intervention materials, as well as emotional exhaustion and reduced intervention delivery [18, 19, 20].

We kept the compliance predictors consistent between models as there was insufficient theory or evidence to justify variable rotation. This was therefore classified as a type N decision.

Accordingly, ten theoretically plausible specifications were identified and pre-registered (<https://osf.io/z62wa>).

**References**

1. Steegen S, Tuerlinckx F, Gelman A, Vanpaemel W. Increasing Transparency Through a Multiverse Analysis. Perspect Psychol Sci. 2016;11(5):702-12, doi:10.1177/1745691616658637.

2. Simonsohn U, Simmons JP, Nelson LD. Specification curve: Descriptive and inferential statistics on all reasonable specifications. Available at SSRN 2694998. 2019.

3. Simonsohn U, Simmons JP, Nelson LD. Specification curve analysis. Nature Human Behaviour. 2020;4(11):1208-14, doi:10.1038/s41562-020-0912-z.

4. Del Giudice M, Gangestad SW. A traveler’s guide to the multiverse: Promises, pitfalls, and a framework for the evaluation of analytic decisions. Advances in Methods and Practices in Psychological Science. 2021;4(1):1-15, doi:10.1177/2515245920954925.

5. Short C, Breznau N, Bruntsch M, Burkhardt M, Busch N, Cesnaite E, et al. Multi-Curious: A Multi-Disciplinary Guide To Multiverse Analysis. pre-print.

6. O’Brien A, Hamilton S, Humphrey N, Qualter P, Boehnke JR, Santos J, et al. Examining the impact of a universal social and emotional learning intervention (Passport) on internalising symptoms and other outcomes among children, compared to the usual school curriculum: study protocol for a school-based cluster randomised trial. Trials. 2023;24(1):703, doi:10.1186/s13063-023-07688-0.

7. Rojas-Andrade R, Bahamondes LL. Is implementation fidelity important? A systematic review on school-based mental health programs. Contemporary School Psychology. 2019;23(4):339-50, doi:10.1007/s40688-018-0175-0.

8. O’Brien A, Panayiotou M, Santos J, Hamilton S, Humphrey N. A systematic review informing recommendations for assessing implementation variability in universal, school-based social and emotional learning interventions. Social and Emotional Learning: Research, Practice, and Policy. 2025;5:100112, doi:10.1016/j.sel.2025.100112.

9. Peugh JL, Strotman D, McGrady M, Rausch J, Kashikar-Zuck S. Beyond intent to treat (ITT): A complier average causal effect (CACE) estimation primer. Journal of school psychology. 2017;60:7-24, doi:10.1016/j.jsp.2015.12.006.

10. Panayiotou M, Humphrey N, Hennessey A. Implementation matters: Using complier average causal effect estimation to determine the impact of the Promoting Alternative Thinking Strategies (PATHS) curriculum on children’s quality of life. Journal of Educational Psychology. 2020:236-53, doi:10.1037/edu0000360.

11. Bradshaw CP, Shukla KD, Pas ET, Berg JK, Ialongo NS. Using complier average causal effect estimation to examine student outcomes of the PAX Good Behavior Game when integrated with the PATHS curriculum. Administration and Policy in Mental Health and Mental Health Services Research. 2020;47:972-86, doi:10.1007/s10488-020-01034-1.

12. Berg JK, Bradshaw CP, Jo B, Ialongo NS. Using complier average causal effect estimation to determine the impacts of the good behavior game preventive intervention on teacher implementers. Administration and Policy in Mental Health and Mental Health Services Research. 2017;44(4):558-71, doi:10.1007/s10488-016-0738-1.

13. Ashworth E, Panayiotou M, Humphrey N, Hennessey A. Game On—Complier Average Causal Effect Estimation Reveals Sleeper Effects on Academic Attainment in a Randomized Trial of the Good Behavior Game. Prevention Science. 2020;21(2):222-33, doi:10.1007/s11121-019-01074-6.

14. Humphrey N, Panayiotou M, Hennessey A, Ashworth E. Treatment effect modifiers in a randomized trial of the good behavior game during middle childhood. Journal of Consulting and Clinical Psychology. 2021;89(8):668, doi:10.1037/ccp0000673.

15. Humphrey N, Panayiotou M. Bounce Back: randomised trial of a brief, school-based group intervention for children with emergent mental health difficulties. European Child & Adolescent Psychiatry. 2022;31(1):205-10, doi:10.1007/s00787-020-01612-6.

16. Domitrovich CE, Bradshaw CP, Poduska JM, Hoagwood K, Buckley JA, Olin S, et al. Maximizing the implementation quality of evidence-based preventive interventions in schools: A conceptual framework. Advances in school mental health promotion. 2008;1(3):6-28, doi:10.1080/1754730x.2008.9715730.

17. Humphrey N, Hennessey A, Lendrum A, Wigelsworth M, Turner A, Panayiotou M, et al. The PATHS curriculum for promoting social and emotional well-being among children aged 7–9 years: a cluster RCT. 2018, doi:10.3310/phr06100.

18. Musci RJ, Pas ET, Bettencourt AF, Masyn KE, Ialongo NS, Bradshaw CP. How do collective student behavior and other classroom contextual factors relate to teachers’ implementation of an evidence-based intervention? A multilevel structural equation model. Development and Psychopathology. 2019;31(5):1827-35, doi:10.1017/S095457941900097X.

19. Domitrovich CE, Pas ET, Bradshaw CP, Becker KD, Keperling JP, Embry DD, et al. Individual and school organizational factors that influence implementation of the PAX good behavior game intervention. Prevention Science. 2015;16(8):1064-74, doi:10.1007/s11121-015-0557-8.

20. Swift LE, Hubbard JA, Bookhout MK, Grassetti SN, Smith MA, Morrow MT. Teacher factors contributing to dosage of the KiVa anti-bullying program. Journal of School Psychology. 2017;65:102-15, doi:10.1016/j.jsp.2017.07.005.
