# Supplementary material for: CACE closed: A multiverse examination of the influence of implementation variability on student outcomes in a randomised controlled trial of a universal, school-based social-emotional learning intervention
Source: PLoS One. 2026 Jun 2;21(6):e0349949. doi: 10.1371/journal.pone.0349949 (PMC13229310; doi:10.1371/journal.pone.0349949)
Supplement: S3 File — (DOCX) [file pone.0349949.s003.docx]

**CACE closed: A multiverse examination of the influence of implementation variability on student outcomes in a randomised controlled trial of a universal, school-based social-emotional learning intervention**

**Confirmatory factory analysis.** Traditional model fit indices, alongside factor loadings and communalities, and internal consistency coefficients are reported in Tables S2 and S3. The Peer support model was found to have an acceptable model fit, but model saturation was observed for loneliness and victimisation; conclusions about model fit could therefore not be reached, however factor loadings were deemed to be acceptable.

Model fit across compliance proxies was acceptable (Table S3). Comparatively lower model fit was observed for quality of delivery; closer inspection revealed that two items had factor loadings below .80. These items were: (1) I felt able to respond to the needs of children during Passport activities (λ = .65) and (2) I felt well prepared to teach Passport lessons (λ = .7). Given the substantial loadings across all factors, and generally acceptable fit, we proceeded with the CACE as planned. Nonetheless sample size was small and psychometric testing should be replicated with a larger sample.

**Table S1.  Teacher implementation survey**

|  |  |  |  |  |  |  |  |  |  |
| --- | --- | --- | --- | --- | --- | --- | --- | --- | --- |
| **Implementation dimension** | **Items** | **Response format** | **Scoring** | **Factor loading** | **RMSEA**  **(90% CI)** | **CFI** | **SRMR** | **χ²** | **Alpha** |
| Dosage |  | Yes/No | Sum score |  |  |  |  |  |  |
|  | 1 Did you teach Session 0: Beginning the adventure? |  |  |  |  |  |  |  |  |
|  | 2 Did you teach Session 1: Valuing our differences and similarities |  |  |  |  |  |  |  |  |
|  | 3 Did you teach Session 2: Understanding and expressing our emotions |  |  |  |  |  |  |  |  |
|  | 4 Did you teach Session 3: Recognising other people’s feelings |  |  |  |  |  |  |  |  |
|  | 5 Did you teach Session 4: Helping each other and coping skills |  |  |  |  |  |  |  |  |
|  | 6 Did you teach Session 5: Friendship |  |  |  |  |  |  |  |  |
|  | 7 Did you teach Session 6: Challenges in friendship |  |  |  |  |  |  |  |  |
|  | 8 Did you teach Session 7: Dealing with frustration |  |  |  |  |  |  |  |  |
|  | 9 Did you teach Session 8: Dealing with stress |  |  |  |  |  |  |  |  |
|  | 10 Did you teach Session 9: Dealing with conflict |  |  |  |  |  |  |  |  |
|  | 11 Did you teach Session 10: Unfairness in daily life |  |  |  |  |  |  |  |  |
|  | 12 Did you teach Session 11: Dealing with bullying |  |  |  |  |  |  |  |  |
|  | 13 Did you teach Session 12: Unfairness and injustice in the world |  |  |  |  |  |  |  |  |
|  | 14 Did you teach Session 13: Coping with change |  |  |  |  |  |  |  |  |
|  | 15 Did you teach Session 14: Coping with loss |  |  |  |  |  |  |  |  |
|  | 16 Did you teach Session 15: Helping others in difficult situations |  |  |  |  |  |  |  |  |
|  | 17 Did you teach Session 16: Let’sreview |  |  |  |  |  |  |  |  |
|  | 18 Did you teach Session 17: Celebration |  |  |  |  |  |  |  |  |
| Fidelity | For the following items, we want you to think about your use of the Passport guidance materials when teaching the above lessons: | Slider, 0-100% | Average*dosage |  | 0 | 1 | 0 | (3) = 54.3, p < .001 | 0.84 |
|  | 1 I covered the key goals/objectives of Passport lessons that I taught |  |  | .77 |  |  |  |  |  |
|  | 2 I followed the structure and sequence of activities outlined in the guidance materials for Passport lessons that I taught |  |  | .83 |  |  |  |  |  |
|  | 3 I adhered to the guidance materials when teaching the core activities of Passport lessons |  |  | .82 |  |  |  |  |  |
| Quality | For the following items, we want you to think about how you felt about teaching the above lessons: | Slider, 0-100% | Average*dosage |  | .297 (.134, .489) | .816 | .048 | χ²(6) = 49.98 | 0.84 |
|  | I felt able to clearly explain key activities to children when teaching Passport lessons |  |  | .81 |  |  |  |  |  |
|  | I felt able to respond to the needs of children during Passport activities |  |  | .65 |  |  |  |  |  |
|  | I felt able to engage the pupils in my class during Passport lessons |  |  | .87 |  |  |  |  |  |
|  | I felt well prepared to teach Passport lessons |  |  | .7 |  |  |  |  |  |
| Responsiveness | For the following items, we want you to think about how children in your class responded when you were teaching the above lessons: | Slider, 0-100% | Average*dosage |  | .224 (.049, .424) | .953 | .025 | χ²(6) = 104.97, p < .001 | 0.94 |
|  | Children in my class actively participated(e.g., initiated and elaborated on discussion topics) in Passport |  |  | .76 |  |  |  |  |  |
|  | Children in my class engaged (e.g., completed activities with interest) with Passport |  |  | .93 |  |  |  |  |  |
|  | Children in my class showed interest and enthusiasm (e.g., looked forward to lessons, asked/reminded you about them) for Passport |  |  | .99 |  |  |  |  |  |
|  | Children in my class enjoyed (e.g., discussed lessons positively) taking part in Passport |  |  | .94 |  |  |  |  |  |
| Reach | For the following item, we want you to think about who was present when you were teaching the above lessons: | Slider, 0-100% | Average*dosage |  |  |  |  |  |  |
|  | 1 What proportion of your class that were present, on average, during Passport lessons? *Consider withdrawal for additional support etc.* |  | |  |  |  |  |  |  |

**Table S2. Confirmatory factor analysis results for pupil outcome variables.**

| Variable | Item | Factor loading | RMSEA (90% CI) | CFI | SRMR | χ² | h^2^range | Cronbach’s alpha |
| --- | --- | --- | --- | --- | --- | --- | --- | --- |
| Peer support |  |  | .051 (.028, .078) | .99 | .015 | χ²(6) = 1128.67, p < 0.001 | .31, .39 | 0.71 |
|  | 1 Spent time with friends | .6 |  |  |  |  |  |  |
|  | 2 Had fun with friends | .7 |  |  |  |  |  |  |
|  | 3 Helped each other | .54 |  |  |  |  |  |  |
|  | 4 Been able to rely on friends | .63 |  |  |  |  |  |  |
| Peer victimisation |  |  | 0 | 1 | 0 | χ²(3) = 845.95, p < 0.001 | .24, .68 | 0.75 |
|  | 1 Afraid of others | .5 |  |  |  |  |  |  |
|  | 2 Made fun of by others | .81 |  |  |  |  |  |  |
|  | 3 Been bullied by others | .82 |  |  |  |  |  |  |
| Lonely |  |  | 0 | 1 | 0 | χ²(3) = 1001.47, p < 0.001 | .34, .55 | 0.71 |
|  | 1 Feel like no one to talk to | .59 |  |  |  |  |  |  |
|  | 2 Feel left out | .7 |  |  |  |  |  |  |
|  | 3 Feel alone | .72 |  |  |  |  |  |  |
