# Supplementary material for: CACE closed: A multiverse examination of the influence of implementation variability on student outcomes in a randomised controlled trial of a universal, school-based social-emotional learning intervention
Source: PLoS One. 2026 Jun 2;21(6):e0349949. doi: 10.1371/journal.pone.0349949 (PMC13229310; doi:10.1371/journal.pone.0349949)
Supplement: Attachment — Submitted filename: PLOS ONE - Appeal Request.docx [file pone.0349949.s006.docx]

The Ellen Wilkinson Building

The University of Manchester

Oxford Road

Greater Manchester

2.12.2025

Dear Prof Sarmiento,

I am writing to formally appeal the decision to reject our manuscript, titled *CACE closed: A multiverse examination of the influence of implementation variability on student outcomes in a randomised controlled trial of a universal, school-based social-emotional learning intervention* (PONE-D-25-61187).

My co-authors and I sincerely thank you for the time and effort dedicated to reviewing our submission. We are disappointed by the decision not to send our paper for external peer review, which we believe stems from a misunderstanding of our analytic approach. We wish to clarify that our use of sensitivity analysis with pre-specified compliance thresholds is distinct from post-hoc data exploration and aligns with recommended practices in educational intervention research and recent publications in PLOS ONE. Below, we provide a point-by-point response detailing our rationale for reconsidering the decision to reject this manuscript prior to external peer review.

**Comment 1:** *Allowing researchers to define multiple compliance thresholds could lead to spurious results and reduce study power by testing multiple hypotheses.*

**Response:** We appreciate this methodological concern but believe it stems from a misunderstanding of our analytic approach. Our study adopts a pre-registered multiverse framework (https://osf.io/s5pmw/overview) specifically designed to address the problem of spurious results arising from researcher degrees of freedom in defining and modelling compliance.

First, we address the concern about defining two compliance thresholds.

CACE is limited by its reliance on a binary marker of compliance because defining 'full' compliance of a prevention intervention is significantly challenging. CACE is dependent on the exclusion restriction assumption (non-compliers derive no benefit from the intervention) which is difficult to validate in the absence of theory-informed compliance thresholds (Education Endowment Foundation, 2018) which are largely untested and unknown in psychological and educational interventions (Durlak, 2010). Accordingly, Peugh and colleagues (2017) recommend: "*a series of additional sensitivity analyses could be conducted using several binary compliance indicator (“U”) variables defined at multiple cut-points (e.g., 50%, 70%, 90%, 95%) and observing the change in the CACE treatment effect estimate”* (p.23).

We adopt this advice, but rather than making a single arbitrary choice when defining ‘full’ compliance and subsequently presenting misleading results, we pre-registered a sensitivity analysis examining two compliance thresholds (50^th^ and 75^th^ percentile). This choice follows established practice in educational research (e.g., Ashworth et al., 2020; Berg et al., 2017; Bradshaw et al., 2020; Humphrey & Panayiotou, 2022; Humphrey et al., 2021; Panayiotou et al., 2020). This decision was also pragmatic, as each CACE model requires substantial computational time (>24 hours) and it will facilitate comparison with other intervention studies using identical thresholds, such as the ones cited above.

Our pre-registration details the above rationale, which we hope instils in the Editor our recognition of the importance of this discussion. This is signposted on page 17 (lines 405, 11 - 415) of our manuscript, detailed in a supplementary file (File S2) and reported in our preregistration (<https://osf.io/s5pmw/overview>).

Second, we address the concern about reducing study power by testing multiple hypotheses briefly here, and in more detail below.

A correction for multiplicity was not applied in the current study as the multiverse is exploratory: the goal is not to correcting for multiplicity (i.e., alpha adjustment) but to increase transparency by visualising the distribution of outcomes. This is reported in the manuscript on page 19 lines 433-436.

**Action taken:** We have revised the introduction (page 8 lines 192-202) and the methods (page 17 lines 405, 11 - 415). These sections now discuss the challenge in defining full compliance in prevention interventions, report the recommendation to carry out sensitivity analyses and cites empirical research demonstrating this practice in education. Should this manuscript be reconsidered, and further detail be required, we are happy to be guided on this.

**Comment 2:** *Although formal approaches to assess implementation variability are valuable, the manuscript risks creating misunderstandings about sensitivity analysis and its role in research.*

**Response:** We take this concern seriously and want to ensure that our work contributes to methodological clarity rather than obscures it.

PLOS ONE has championed the use of multiverse analysis to assess robustness across analytical decisions (e.g., Claesen et al., 2023; Dürlinger et al., 2022) and highlighted how such approaches remain underutilised (e.g., Böschen, 2023). Our study extends the application of this framework to school-based intervention research to examine whether conclusions remain stable when there are a range of reasonable ways to define compliance. We report on page 22 (lines 519-522) that the use of a multiverse framework is not to discover which model conditions result in desirable outcomes (i.e., cherry picking), but whether results are robust to these variations.

Our pre-registered multiverse framework aims to address, rather than exacerbate, concerns about multiple testing by transparently reporting pragmatic and plausible specifications. This differs from post-hoc specification searching, selective reporting, or testing multiple hypothesis without adjustment. A multiverse analysis framework does not inherently require an alpha adjustment for multiplicity because its primary goal is to assess the robustness of a finding across all plausible analytical paths, rather than to make a single, definitive confirmatory claim based on a chosen one (Clayson, 2024). The exploratory nature of these analyses are noted on page 19, lines 424-6 of our manuscript.

We recognise that our manuscript may benefit from clearer articulation of when such analytic approaches are appropriate and the inferential limitations. Should our manuscript be reconsidered, we would welcome specific guidance on where our presentation risks creating misunderstandings and would revise accordingly.

Thank you for your time and consideration of our request. We look forward to hearing from you.

Best wishes,

Annie O’Brien, on behalf of the authorship team

**References**

Ashworth, E., Panayiotou, M., Humphrey, N., & Hennessey, A. (2020). Game On—Complier Average Causal Effect Estimation Reveals Sleeper Effects on Academic Attainment in a Randomized Trial of the Good Behavior Game. Prevention Science, 21(2), 222-233. <https://doi.org/10.1007/s11121-019-01074-6>

Berg, J. K., Bradshaw, C. P., Jo, B., & Ialongo, N. S. (2017). Using complier average causal effect	estimation to determine the impacts of the good behavior game preventive intervention on teacher implementers. Administration and Policy in Mental Health and Mental Health Services Research, 44(4), 558-571. <https://doi.org/10.1007/s10488-016-0738-1>

Böschen I (2023) Changes in methodological study characteristics in psychology between 2010-2021.	PLOS ONE 18(5): e0283353.<https://doi.org/10.1371/journal.pone.0283353>

Bradshaw, C. P., Shukla, K. D., Pas, E. T., Berg, J. K., & Ialongo, N. S. (2020). Using complier average causal effect estimation to examine student outcomes of the PAX Good Behavior Game when integrated with the PATHS curriculum. Administration and Policy in Mental Health and Mental Health Services Research, 47, 972-986. <https://doi.org/10.1007/s10488-020-01034-1>

Claesen A, Vanpaemel W, Maerten AS, Verliefde T, Tuerlinckx F, et al. (2023) Data sharing upon request and statistical consistency errors in psychology: A replication of Wicherts, Bakker and Molenaar (2011). PLOS ONE 18(4): e0284243. <https://doi.org/10.1371/journal.pone.0284243>

Clayson, P. E. (2024). Beyond single paradigms, pipelines, and outcomes: Embracing multiverse analyses in psychophysiology. *International Journal of Psychophysiology*, *197*, 112311. <https://doi.org/https://doi.org/10.1016/j.ijpsycho.2024.112311>

Durlak, J. A. (2010). The importance of doing well in whatever you do: A commentary on the special section, “Implementation research in early childhood education”. Early childhood research quarterly, 25(3), 348-357. [https://doi.org/https://doi.org/10.1016/j.ecresq.2010.03.003](https://doi.org/https:/doi.org/10.1016/j.ecresq.2010.03.003)

Dürlinger, F., & Pietschnig, J. (2022). Meta-analyzing intelligence and religiosity associations: Evidence from the multiverse. Plos one, 17(2), e0262699. <https://doi.org/10.1371/journal.pone.0262699>

EEF. (2018). Statistical analysis guidance for EEF evaluations. Retrieved from <https://educationendowmentfoundation.org.uk/public/files/Evaluation/Writing_a_Protocol_or_SAP/EEF_statistical_analysis_guidance_2018.pdf>

Humphrey, N., & Panayiotou, M. (2022). Bounce Back: randomised trial of a brief, school-based group intervention for children with emergent mental health difficulties. European Child & Adolescent Psychiatry, 31(1), 205-210. <https://doi.org/10.1007/s00787-020-01612-6>

Humphrey, N., Panayiotou, M., Hennessey, A., & Ashworth, E. (2021). Treatment effect modifiers in a randomized trial of the good behavior game during middle childhood. Journal of consulting and clinical psychology, 89(8), 668. <https://doi.org/10.1037/ccp0000673>

Panayiotou, M., Humphrey, N., & Hennessey, A. (2020). Implementation matters: Using complier average causal effect estimation to determine the impact of the Promoting Alternative Thinking Strategies (PATHS) curriculum on children’s quality of life [doi:10.1037/edu0000360]. Journal of Educational Psychology, 236-253. <https://doi.org/10.1037/edu0000360>

Peugh, J. L., Strotman, D., McGrady, M., Rausch, J., & Kashikar-Zuck, S. (2017). Beyond intent to treat (ITT): A complier average causal effect (CACE) estimation primer. Journal of school psychology, 60, 7-24. <https://doi.org/10.1016/j.jsp.2015.12.006>
