# Supplementary material for: CACE closed: A multiverse examination of the influence of implementation variability on student outcomes in a randomised controlled trial of a universal, school-based social-emotional learning intervention
Source: PLoS One. 2026 Jun 2;21(6):e0349949. doi: 10.1371/journal.pone.0349949 (PMC13229310; doi:10.1371/journal.pone.0349949)
Supplement: Attachment — Submitted filename: PLOS One multiverse reviewer comments 03.26.docx [file pone.0349949.s007.docx]

We thank the reviewers for their careful reading of the manuscript and for their constructive and thoughtful comments. We have responded to each comment in turn below and have revised the manuscript accordingly where appropriate. We believe that these revisions have strengthened the clarity, rigour, and contribution of the paper, and we are grateful for the opportunity to address the reviewers’ feedback.

**Reviewer #1**

**In their manuscript “CACE closed: A multiverse examination of the influence of implementation variability on student outcomes in a randomised controlled trial of a universal, school-based social-emotional learning intervention”, the authors report a preregistered secondary analysis of a large randomised control trial evaluating a school-based social-emotional learning intervention (Passport). Notably, the authors apply a preregistered multiverse analysis to complier average causal effect modelling to report whether results on relational outcomes are robust to defensible analytical flexibility regarding how compliance is defined and thresholded. Across their multiverse of 27 pipelines, the authors report consistently null effects, with one isolated statistically significant negative effect for peer support under a high-reach specification. The central conclusion is that substantive inferences about intervention effectiveness are sensitive to researcher degrees of freedom in CACE modelling, and that a multiverse approach offers a transparent way to report robustness in implementation science.

I would like to begin by expressing support for the application of a multiverse framework. A growing body of work across scientific disciplines has demonstrated that defensible analytic flexibility can influence the reported results, and selective reporting of a single analytic pipeline risks overstating confidence in conclusions. I believe that the multiverse analysis approach offers a systematic and comprehensive framework to report the robustness of results across researcher degrees of freedom. It is encouraging to see this approach applied to compliance-adjusted casual inference in educational research, and the theoretical motivation for this application is well-grounded in the manuscript.

The manuscript has several additional strengths. The analyses are preregistered, the authors appropriately emphasise robustness, entropy and uncertainty rather than statistical significance alone, and the discussion is generally cautious and methodologically reflective.

However, I have identified several major and minor issues that, if addressed, would strengthen the manuscript prior to publication. My expertise is particularly well aligned with the evaluation of the multiverse analysis and its interpretation.**

We wish to express our sincere gratitude for your time, expertise, and words of support for this manuscript. We have taken feedback on board and believe it has significantly strengthened this manuscript and for that we are most thankful.

**Major issue 1: First, the introduction would benefit greatly from restructuring. Currently, it begins with an explanation of multiverse analysis, which is followed by the rationale for its application to the substantive topic of the manuscript. I believe clarity would be improved by first introducing the substantive problem, and then presenting multiverse analysis as a methodological solution for transparently reporting uncertainty. As written, the opening gives the impression that the manuscript primarily advances multiverse analysis as a method, rather than using it as a tool to address a specific substantive question.**

Thank you for this helpful comment – upon revisiting the manuscript, we agree that restructuring the introduction would improve the clarity of our aims as well as the readability of our manuscript.

We have restructured the introduction as requested.

**Second, the manuscript occasionally overstates what multiverse analysis achieves. For example, the authors suggest that multiverse analysis can be used to ‘limit researcher degrees of freedom’. In its current application, the multiverse analysis does not identify superior pipelines from those computed. Rather, it documents how results vary across defensible choices. In this sense, multiverse analysis reports uncertainty arising from researcher degrees of freedom rather than constraining them. The role of multiverse analysis as a means to report uncertainty is explained within this paper by Hoffmann and colleagues:

Hoffmann, S., Schönbrodt, F., Elsas, R., Wilson, R., Strasser, U., & Boulesteix, A. L. (2021). The multiplicity of analysis strategies jeopardizes replicability: lessons learned across disciplines. Royal Society Open Science, 8(4), 201925.** [**https://doi.org/10.1098/rsos.201925 [doi.org]**](https://urldefense.com/v3/__https:/doi.org/10.1098/rsos.201925__;!!PDiH4ENfjr2_Jw!A0O--ynU4arSS86klOR6Ln7KM8ZaF5dUCKO6YJKileTm2iD_vpy40SgPpcQG4MUK0qEq7auuuv5IPXV23oyTyEVkIQ$) **And a recent community opinion on what multiverse analysis can and cannot address is presented in a preprint, along with guidelines for its implementation:

Short, C. A., Breznau, N., Bruntsch, M., Burkhardt, M., Busch, N., Cesnaite, E., ... & Hildebrandt, A. (2025). Multi-curious: A multi-disciplinary guide to a multiverse analysis. MetaArXiV.** [**https://doi.org/10.31222/osf.io/4yzeh_v2 [doi.org]**](https://urldefense.com/v3/__https:/doi.org/10.31222/osf.io/4yzeh_v2__;!!PDiH4ENfjr2_Jw!A0O--ynU4arSS86klOR6Ln7KM8ZaF5dUCKO6YJKileTm2iD_vpy40SgPpcQG4MUK0qEq7auuuv5IPXV23ow11pfMtg$) **In lines 221-222, the authors state that ‘researcher degrees of freedom still limit this method’. I wonder whether it would be more accurate to say that unreported researcher degrees of freedom is rather limiting replicability and, when unreported, leads to uncertainty. This my be more conceptually precise than arguing that researcher degrees of freedom limits the method.**

Thank you for sharing this paper and preprint – they have both been enjoyable reads and are thought provoking.

We understand the concern raised about how current phrasing overstates what it is that the multiverse can and cannot achieve. We have clarified our reporting of the aims and outcomes of the multiverse analysis. For examples, please see:

- Page 2 lines 36-48
- Page 10 lines 234-250
- Page 11 lines 270-271
- Page 33 lines 809-812

**Third, as many readers may not yet be familiar with the multiverse analysis framework, the manuscript would benefit from a concise, explicit statement, ideally in the methods or results section, clarifying the claims that can and cannot be made based on the multiverse analysis presented (e.g., descriptive robustness report rather than identifying superior analytical decisions). This may prevent the assumption that the pipeline producing a significant effect is methodologically superior to the other pipelines, which cannot be deduced from the present results.**

Thank you for raising this - we have expanded upon our note of caution in the discussion section:

Page 25 lines 566-572: We recommend caution when interpreting the results of the multiverse, which does not identify superior analytic decisions based on, for example, statistical significance, rather it provides a descriptive report of the robustness and replicability of results [12]. In other words, its purpose is not to discover which model conditions result in desirable outcomes (i.e., cherry picking), but transparently report whether results are robust to these variations.

**Fourth, greater transparency is needed regarding how the set of analytic options included in the multiverse was identified. The authors cite Del Giudice and Gangestad (2021) as guiding the construction of the multiverse, but this framework primarily concerns equivalence among pipelines rather than procedures for identifying candidate options in the first place. While Supplementary File 2 provides additional detail, some justifications (for example, excluding options because ‘both specifications are known to result in a different outcome’) are unclear. Different outcomes of which metric, and why this constitutes grounds for exclusion? Multiverse analyses have previously identified analytic options through approaches such as systematic literature review, crowdsourcing, or expert consensus. The authors should clearly report where the pool of candidate options originated, how inclusion and exclusion decisions were made, and what procedure led to the final set of pipelines. Adopting the structured guidance proposed by Short et al. (2025) may help strengthen transparency, allow readers to evaluate potential bias, and aid reproducibility.**

We have revised File S2. This file now contains detailed decisions about how we identified candidate options for the multiverse, the available options, and the decisions made. This now includes explicit reference to our systematic review and crowdsourcing approach.

**Fifth, there is considerable repetition throughout the manuscript, including within individual paragraphs. For example, in the Statistical Analysis section (beginning on line 420), the justification for using two-level models with SE adjustment is repeated across successive sentences. In particular, the rationale given in the third and fifth sentences of this paragraph could be combined to convey the same information more concisely. Similar repetition occurs elsewhere in the manuscript, and reducing it would substantially improve clarity and readability without loss of content.**

We have amended this section as requested. We have also reviewed the manuscript and made changes throughout to reduce repetition. For example:

- Page 8 lines 176-186
- Page 12 lines 264-281
- Page 17-18 lines 384-388
- Page 18 lines 392-403

**Minor issues: Sixth, the multiverse plots may be more visually informative if presented in a specification curve style format. A visual summary of the pipelines would also be helpful, to quickly deduce which options and combinations of options were included in the multiverse analysis. This could be in the format of a sankey plot.

See specification curve plot here: Simonsohn, U., Simmons, J. P., & Nelson, L. D. (2020). Specification curve analysis. Nature Human Behaviour, 4(11), 1208-1214.** [**https://doi.org/10.1038/s41562-020-0912-z [doi.org]**](https://urldefense.com/v3/__https:/doi.org/10.1038/s41562-020-0912-z__;!!PDiH4ENfjr2_Jw!A0O--ynU4arSS86klOR6Ln7KM8ZaF5dUCKO6YJKileTm2iD_vpy40SgPpcQG4MUK0qEq7auuuv5IPXV23ow4L6_4gQ$)

Thank you for these suggestions.

We believe that an advantage of the existing figures is that they are simple, intuitive and accessible to those unfamiliar with multiverse analysis and specification curve analysis. However, upon review of our figures we do think that they could be improved for readability. We therefore have reduced the length of the x-axis for each figure (see updated Figures 2-4).

We agree that it would be helpful to include a visual illustration of the multiverse pipelines. As Sankey plots are commonly used for longitudinal data, we thought it best to use a tree diagram to visually illustrate this information. We have depicted this in a new figure (Figure 1).

**Seventh, I am not sure that the elephant analogy adds clarity or scientific meaning (e.g., referring to entropy values as ‘the elephant’s trunk’, precision and confidence intervals as 'the elephant's tusk’, direction and effect sizes as ‘the elephant’s tail’ etc.). For readers new to multiverse analysis, I think this analogy may add to the cognitive load for readers unfamiliar with multiverse analysis and potentially cause confusion. I understand this is perhaps personal preference, but I would prefer direct explanations.**

This analogy has been removed from the manuscript.

**Finally, the authors should complete a final proof reading of the manuscript to correct mistakes such as un-paired brackets in the text (e.g., line 156) and double commas (e.g., line 208).**

Thank you for spotting these punctuation mistakes which have now been corrected. We have also reviewed the manuscript and corrected remaining grammatical errors.

**Overall, I believe this is a methodologically rigourous, well-motivated and transparent study that fits within PLOS ONE’s publication criteria. Addressing the points above would substantially strengthen clarity and guard against misinterpretation.**

Thank you for your constructive and supportive feedback on our manuscript. We have implemented the requested changes and believe that the manuscript has been significantly strengthened as a result.

**Reviewer 2**

**The initial criticism regarding "spurious results" and "reduced study power" appears to conflate traditional confirmatory hypothesis testing with Multiverse Analysis. The manuscript employs a pre-registered multiverse framework (OSF), which fundamentally mitigates the risks of p-hacking or post-hoc data dredging. By testing 10 pre-specified compliance (CACE) models across distinct theoretical dimensions (such as fidelity, dosage, and reach), the authors are not "cherry-picking" a p-value; rather, they are mapping the robustness and stability of the intervention effect against the inherent "researcher degrees of freedom" that often plague school-based trials.

From a causal inference perspective, defining a "complier" in a real-world educational setting is a notorious methodological challenge. The authors' decision to apply multiple thresholds (50th and 75th percentiles) aligns with cutting-edge recommendations from scholars like Peugh et al. (2017) and institutions such as the Education Endowment Foundation. This transparency is essential for validating the exclusion restriction assumption of the CACE model, ensuring that the findings regarding the "Passport" intervention are not merely artifacts of a single, arbitrary statistical cutoff.

Ultimately, this work represents an exemplary application of rigorous, open science that fits perfectly within the scope of PLOS ONE. It provides a valuable contribution to implementation science by demonstrating how varying compliance metrics can shift, or confirm, an effect.**

We extend our sincerest thanks to Reviewer 2 for their encouraging feedback on our manuscript.

**Reviewer #3**

**1. It is noted that the statistical analysis of the tables attached in Supplementary File 2 was not adequately addressed. For example the significance of the R2 value, its significance, and the justification for its decrease in some cases. Likewise, the analysis of what was stated in Supplementary File 3 suffers from a lack of depth in expansion and statistical significance.**

File S1: We have changed R2 to lamba in File S2. We do not discuss the variation in this value as it does not impact our models or results, nor is it relevant to our research question. As this manuscript is already of considerable length, with detailed discussion of model variations, we feel that further discussion of this offers little value to the reader.

File S2: This file has 12 tabs detailing the output of the multiverse analysis. Statistical significance is denoted by an asterisk, which is explained under each table. The model results, and variation in results, are discussed in depth throughout the results and discussion sections of the manuscript.

For example, we examine, identify and discuss the presence and absence of statistically significant compliance predictors across models (page 27 line 609-622) and we discuss the implications of this finding for future research (page 33 lines 774-782).

To improve clarity within the tables, we have changed the title of the teacher-level variables – for example ‘SEL’ has been changed to ‘Attitudes towards SEL’.

**2. It was suggested that a 50% threshold would suffice for the compliance standard.**

This misunderstanding stems from a grammatical error, as our pre-registration states that a 50% and 75% threshold would be implemented. For clarity, we have changed the order of sentences in the Introduction (page 10 lines 230-234) to read:

*Researchers are encouraged to use multiple sensitivity analyses to observe any change across compliance thresholds [46]. Using the 50^th^ and 75^th^ percentile to define compliance is established practice in educational research [41, 49, 50, 57, 58].*

**3. I believe that analyzing sensitivity is appropriate for the study.**

We appreciate that the reviewer agrees with our analysis – thank you.
